# Advanced EUS Guided Tissue Acquisition Methods for Pancreatic Cancer

**DOI:** 10.3390/cancers10020054

**Published:** 2018-02-17

**Authors:** Pujan Kandel, Michael B. Wallace

**Affiliations:** Department of Gastroenterology and Hepatology Mayo Clinic Florida, 4500 San Pablo Road, Jacksonville, FL 32224, USA; kandel.pujan@mayo.edu

**Keywords:** pancreas cancer, endoscopic ultrasound, precision therapy

## Abstract

Pancreas cancer is a lethal cancer as the majority patients are diagnosed at an advanced incurable stage. Despite improvements in diagnostic modalities and management strategies, including surgery and chemotherapies, the outcome of pancreas cancer remains poor. Endoscopic ultrasound (EUS) is an important imaging tool for pancreas cancer. For decades, resected pancreas cancer and other cancer specimens have been used to identify tissue biomarkers or genomics for precision therapy; however, only 20% of patients undergo surgery, and thus, this framework is not useful for unresectable pancreas cancer. With advancements in needle technologies, tumor specimens can be obtained at the time of tissue diagnosis. Tumor tissue can be used for development of personalized cancer treatment, such as performing whole exome sequencing and global genomic profiling of pancreas cancer, development of tissue biomarkers, and targeted mutational assays for precise chemotherapy treatment. In this review, we discuss the recent advances in tissue acquisition of pancreas cancer.

## 1. Introduction

### 1.1. Multidisciplinary Approach to Pancreatic Ductal Adenocarcinoma

Pancreas is currently the third leading cause of cancer related death in the United States [1]. It is anticipated to be the second most common cause by 2030 [2,3]. About 50% of patients are diagnosed with metastatic disease at the time of diagnosis [4]. Only 15–20% of patients are eligible for surgical resection, which is the only potential curative treatment at present. However, most patients will also ultimately develop metastatic disease, even if they are diagnosed and treated when they are in the earlier stages. The five-year median survival for pancreas cancer is 8% [5]. The shorter survival period and poor outcomes are also due to a lack of diagnostic modalities to find early disease, its late-stage presentation, and its tendency to develop early metastases.

As only a small fraction of patients are eligible for surgical resection, most of the resectable patients receive chemotherapy and/or radiotherapy owing to its impact on five-year survival (10% with surgery alone vs. 25% with the addition of adjuvant therapy) [6]. Furthermore, there has been growing consideration for adding neoadjuvant chemotherapy for borderline resectable and locally advanced pancreatic ductal adenocarcinoma (PDAC). Recent meta-analysis has demonstrated a 70% resection rate in radiographically resectable PDAC [7,8], and resection after neoadjuvant therapy appears to be safe [9]. However optimal management of borderline and locally advanced PDAC is still controversial [10]. Studies have shown favorable outcome with neoadjuvant chemotherapy on highly selected patients with borderline or locally advanced disease who underwent surgery [11]. The goal of therapy in these two groups is to allow significant tumor response for surgical resection. For metastatic disease, the goal of management is palliative and maximizes the quality of life.

Risk factor identification and early detection for PDAC is crucial. Smoking and obesity are modifiable risk factors. Increasing incidence of PDAC has also been seen in patients older than 65 years, men, and African Americans [12]. Chronic pancreatitis, hereditary pancreatitis, and long-standing diabetes are also known risk factors for PDAC [12,13,14,15]. Sporadic disease has been seen in patients with germline mutations like PLAB2, BRACA2, STK11/KLB1, and p16 [16,17]. There is continued need to find a precise therapy for PDAC, despite having excellent knowledge of risk factors.

There have been improvements in preoperative imaging technologies for early diagnosis at the resectable stage, including pancreas-specific computed tomography (CT), magnetic resonance imaging (MRI), and endoscopic ultrasound (EUS) [3]. These advance imaging technologies allow more selective approaches for timely surgical intervention to improve overall survival.

### 1.2. Imaging Evaluation of Pancreas Cancer

Management of PDAC requires a multidisciplinary approach as decisions of resectability and treatment should be carried through a team of oncologists, radiologists, gastroenterologists, and hepatobiliary surgeons. Imaging with dedicated pancreatic magnetic resonance imaging (MRI) and CT protocols help to precisely stage and determine the resectability status of PDAC. Here we discuss different imaging modalities to evaluate pancreas neoplasms.

## 2. Different Imaging Modalities for Pancreas Cancer

### 2.1. Computed Tomography

CT is the first-line imaging tool to evaluate PDAC, recommended by National Comprehensive Cancer Network guidelines for initial evaluation, widely available, and less expensive. It has a sensitivity of 89–97% for diagnosis of PDAC, but this decreases to 65–75% for smaller lesions (<2 cm) [18]. In 10% of cases, a mass could not be visualized, and the only clue to the presence of PDAC was the abrupt cut off of the pancreatic duct and atrophy of pancreatic tissue [19]. Abdominal CT scan is performed with a rapid injection of iodinated contrast; slices of 3 mm or less should be obtained. At least two dual-phase CT are recommended: one at the pancreatic phase (late arterial) and the other at the portal venous phase with contrast enhancement. Pancreatic phase images are helpful to delineate the primary tumor and arterial invasion, and portal venous phase images are used to evaluate venous involvement and identify liver metastases [20]. Tumors are seen as ill-defined hypo intense masses in the pancreas parenchyma. Coronal and sagittal sections of images at arterial and venous phases increase the sensitivity for local invasion identification. CT is less sensitive in detecting small nodal, peritoneal, and hepatic metastases even with high-quality CT scans. Sensitivity was only 15% for detection of nodal metastases, and when a 5 mm cut off for lymph nodes was used, sensitivity increased to 70%, but specificity dropped to 65% [18,19].

### 2.2. Magnetic Resonance Imaging

MRI offers an advantage in certain situations over CT scan for the evaluation of PDAC, despite CT being considered the first-line imaging modality. It has higher resolution and is more sensitive for detection of non-contour-deforming pancreatic tumors [21]. It has better diagnostic values for evaluation of pancreatic cystic, pancreatic duct, and hepatic lesions as metastatic disease when combined with magnetic resonance cholangiopancreatography. It can also be used when CT is contraindicated in the setting of allergy or renal insufficiency. PDAC appears as a hypo intense lesion compared to surrounding parenchyma on pre-contrast and initial post-contrast MRI. Tumors become isointense and show delayed enhancement in late post-contrast MRI.

### 2.3. Endoscopic Ultrasonography

EUS an important imaging tools for pancreas cancer staging. It is similar to the standard upper endoscopy performed under moderate or deep sedation. It has been reported that EUS has a sensitivity over 95% for detection of benign or malignant pancreatic lesions [22]. It is often highly recommended for screening of high-risk individuals for PDAC alongside MRI [23,24,25,26,27,28]. EUS has benefits over CT or MRI scans for identification of smaller lesions (<20 mm) [29,30]. EUS is recommended for all patients with obstructive jaundice (non-calculus) to identify any pancreatic tumor or other non-neoplastic disease when CT or MRI could not identify lesions. In addition, contrast-enhanced EUS and elastography is often used in the setting of acute pancreatitis, infiltrating cancer, and indwelling biliary stent to increase recognition of pancreas tumor as normal EUS may fail to identify a true pancreas mass [31,32,33,34,35]. However, EUS and contrast-enhanced CT are equivalent in defining the surgical resectability of PDAC [29,30].

### 2.4. EUS Elastography and Contrast-Enhanced EUS in Pancreas Cancer

The heterogeneity of pancreatic cancer may be due to various causes, such as mixture of normal and malignant cell subpopulations within tissue, dense fibroblastic stroma, variations in blood flow, and genetic environment determined by different molecular subtypes [36,37]. Diagnosis of solid pancreatic lesions can be difficult in a background of chronic pancreatitis or in the case of negative or inconclusive sampling. EUS elastography (EUS-E) is an ultrasound technique that measures the hardness of tissues and is useful to measure the level of hardness of solid pancreatic lesions using qualitative scores and/or quantitative methods (strain ratio (SR)) [38]. A recent meta-analysis showed that the sensitivity and specificity of EUS-E in identifying solid pancreatic lesions were 95% and 67%, respectively, when a cut-off value of 6.04 was used to describe the malignant lesions using EUS. In addition, a study by Carrara et al. showed that EUS-E with parenchymal strain (pSR) and fractal-based analysis was useful for evaluation of solid pancreatic lesions [36]. Both wall SR (wSR) and pSR were significantly higher in malignant as compared to benign solid pancreatic lesions (pSR: 24.5 vs. 6.4; *p *< 0.001). Sensitivity, specificity, positive predictive value, negative predictive value and area under the curve were 88.4%, 78.8%, 89.7%, 76.9%, and 86.7%, and 91.3%, 69.7%, 86.5%, 80%, and 85.7%, respectively, with cut-off levels of pSR and wSR at 9.10 and 16.2. This study also showed that the surface fractal dimension (also called fractal analysis) of malignant solid pancreatic lesions, in terms of roughness, was significantly different when compared to that of neuroendocrine tumors and benign solid pancreatic lesions. The European Society of Gastrointestinal Endoscopy (ESGE) recently recommended this imaging technology, stating, “For the differential diagnosis between pancreatic cancer and inflammatory masses, commonly used options include EUS-E, contrast-enhanced harmonic EUS, and repeat sampling” [39]. Thus, EUS-E may play an important role in providing additional imaging evidence in cases of suspected pancreatic cancer where biopsy was inconclusive or failed. 

Contrast-enhanced ultrasound plays a critical role in the diagnosis of solid pancreatic lesions by providing information on tissue microvasculature and perfusion. PDAC appear as hypervascular lesions; inflammatory masses, mainly vascular; and neuroendocrine tumors as a hypervascular pattern. Ultrasound contrast agents consist of small microbubbles that backscatter the ultrasound signal and oscillate in response to sound pressure [40]. New development of linear echoendoscopes and second-generation contrast-enhancement agents have made contrast-enhanced harmonic EUS (CEH-EUS) with significantly higher resolution possible [41]. Recent meta-analysis demonstrated high sensitivity and specificity for diagnosis of PDAC using CEH-EUS [42]. In one study, the diagnostic accuracy of CEH-EUS, EUS-E, and the combination of both for the diagnosis of solid pancreatic tumor was evaluated. Results showed that overall accuracies for determination of malignancy using EUS-E, CEH-EUS, their combination, and EUS-guided tissue acquisition were 98.4% (95% CI: 91.4–99.7), 85.5% (95% CI: 74.7–92.2), 91.9% (95% CI: 82.5–96.5), and 91.5% (95% CI: 83.6–99.5), respectively [40]. CEH-EUS detected malignancy with 89.8% sensitivity and 69.2% specificity with demonstration of a hypo- or hypervascular pattern. Thus, the combination of CEH-EUS and EUS-E may further improve the categorization of solid pancreatic masses by directing to the final diagnosis, mainly to differentiate between autoimmune pancreatitis, chronic pancreatitis, and pancreatic cancer.

### 2.5. EUS-Guided Needle Forceps Biopsy in Pancreas Cancer

EUS-guided FNA (EUS-FNA) is an established technique for tissue acquisition of solid pancreatic lesions. Although diagnosis of malignancy can be made by cytologic specimens obtained by fine needle aspiration (FNA), acquisition of a large histologic core is important in some cases, like autoimmune pancreatitis, lymphoma, and gastrointestinal stromal tumor. In addition, more core tissue can be utilized in next-generation sequencing (NGS) for personalized medicine in PDAC. Due to limitations of flexibility and technical difficulty, use of 19-gauge FNA needles for core tissue acquisition is not often utilized in clinical practice. Recent development of miniature biopsy forceps may enable EUS-guided through-the-needle forceps biopsy (EUS-TTNFB). Results from a previously reported study on procaine models provided potential utility in solid pancreatic lesions [43]. In one study, EUS-TTNFB was used for biopsy of solid pancreatic lesions, utilizing a 0.75 mm biopsy forceps through a 19-gauge FNA needle [44]. A total of 49 passes, a median of three passes per session, were performed. Visible histologic core by EUS-TTNFB was obtained at a rate of 71% per pass. The tissue acquisition rate by EUS-TTNFB alone was 67% per pass and 100% per session; this rate was 94% per pass when EUS-TTNFB and EUS-FNA were combined. With a single pass the accuracy to diagnose malignancy with EUS-TTNFB and EUS-FNA was 83% [44]. No pancreatitis was reported. This study showed that EUS-TTNFB was safe, technically feasible, and could provide histologic specimens in addition to EUS-FNA with a single pass of a 19-gauge needle.

### 2.6. EUS-Guided Confocal Laser Microscopy in Pancreas Cancer

Confocal laser endomicroscopy (CLE) is a unique endoscopic technique that involves imaging of tissue at a subcellular level allowing high magnification of tissue and providing an optical biopsy [45]. In the past few years, a new procedure has been developed called needle-based confocal laser endomicroscopy (nCLE) that includes a mini-CLE probe, which can be passed through a 19-gauge needle during EUS-FNA. nCLE allows real-time visualization of tissue at a microscopic level during endoscopy. Optical needle biopsy may serve as a substitute for histology when significant difficulty arises in tissue acquisition, may help to reduce sampling error as it gives real-time microscopic details, and may also assist endoscopists in confirming adequate tissue acquisition in centers where rapid onsite evaluation (ROSE) is not available. A few studies have reported the feasibility and safety of in vivo imaging of pancreatic masses and have provided the criteria for nCLE characterization of PDAC [46,47]. In one prospective validation study, the diagnostic value and the reproducibility of nCLE criteria was evaluated for solid malignant lesions. Sensitivity, specificity, and accuracy for the nCLE parameter ranged from 19–93%, 0–56%, 26–69% with poor interobserver agreement between the experts *k* = 0.02–0.38 [48]. Thus a diagnostic value of nCLE in solid pancreatic mass is uncertain and the inter-observer agreement even among experts is limited [46]. The main difficulties faced with nCLE include the wide variety of histologic diagnoses, interobserver variability, reproducibility of results, technical issues involved in interpretation of images, sampling error, and the need for endoscopists to learn cytopathologists interpretation [45]. Intense training might increase the inter-and intra-observer variability; however, with the current criteria the reproducibility of CLE parameters is insupportable for application in a clinical practice.

### 2.7. Endoscopic Ultrasound with Fine Needle Aspiration

EUS-FNA is considered the most sensitive imaging tool for evaluation of malignant and benign pancreatic lesions. Lesions are localized with the linear echoendoscope scope and a needle is passed through the accessory channel of the echoendoscope to sample the tumor. The needle is passed in a to-and-from motion for 20–30 s in a fanning pattern to increase the tissue yield. After the needle is withdrawn, all tissue materials are expressed on slides for preparation of thin smears with papanicolaou stain or only core tissues are placed on thin smear slides and in a formalin jar for future pathologic analysis. ROSE by a cytotechnician, when available, provides real-time cytologic diagnosis [49]. This is important because after each needle pass, the on-site cytologists evaluate the adequacy of tissue material obtained and guide whether endoscopists should repeat the FNA. EUS-FNA has a sensitivity of about 85–90% for diagnosis of PDAC, and a specificity of 100% [50]. Complications associated with EUS-FNA are rare, but pancreatitis may occur in about 1% [51]. Another important downside associated with the EUS-FNA technique is a low diagnostic yield (false-negative diagnosis) that may impact patient outcomes [52]. This may occur due to improper EUS-FNA techniques, lesion characteristics, and the experience of endosonographers and cytopathologists, leading to sampling errors [53,54]. The false negative rate for solid pancreas cancer is about 4–45% [53]. The most recent quality indicator by a joint American Society of Gastrointestinal Endoscopy and American College of Gastroenterology (ASGE and ACG) task force has identified the following characteristics in patients undergoing EUS-FNA: (a) diagnostic adequacy for all solid lesions (performance target ≥85%); (b) diagnostic rate and sensitivity for malignancy for pancreas mass (performance target ≥70% and 85%, respectively), and (c) adverse events incidence after EUS-FNA (performance targets are acute pancreatitis: <2%, perforation: <0.5%, and clinically significant bleeding: <1%) [55,56]. Several factors may impact the EUS-FNA outcome, such as: (a) technique; use of suction (wet, dry, and no suction) and stylet, fanning and capillary technique, number of passes (b) needle type (EUS-FNA vs. fine needle biopsy (FNB), needle gauge); (c) endoscopists (training and competency, experience and volume); (d) cytopathologists (training, experience and volume) and expertise of cytotechnician on making slides and cell block) and the availability of ROSE [55].

## 3. Novel Tool and Techniques for EUS-Guided Tissue Acquisition of Pancreas Malignancy

### 3.1. EUS-FNA Tissue Acquisition Technique

There are several techniques available to facilitate adequate sampling of tissue during EUS-FNA.

#### 3.1.1. Use of Stylet

Use of stylet is very common among endoscopists as it is believed that this prevents blockage of needle lumen as it passes through the gastrointestinal wall and target lesion. However, several randomized controlled trials have shown similar performance in specimen adequacy or diagnostic yield of malignancy with or without using a stylet during EUS-FNA [57,58,59]. In addition, results from one randomized controlled trial (RCT) showed that flushing with air slowly is better than second-time insertion of stylet to extract EUS-FNA aspirates of solid pancreatic mass [60]. Suction is also commonly used during EUS-FNA tissue acquisition. In one RCT, 81 patients with pancreatic masses were included and compared with and without using suction during EUS-FNA. Suction groups had higher amounts of cellularity, diagnostic yield, accuracy, and bloodiness [60].

#### 3.1.2. Fanning Technique

The main aim of the fanning technique during EUS-FNA is to procure more tissue. In this technique, the needle is placed at four separate areas within the lesion and moved back and forth several times. The direction of the needle can be changed either with the endoscope up or down. In one RCT of 54 patients with pancreatic masses, the fanning technique was compared with the standard technique to obtain adequate specimen for diagnosis. With the fanning technique, onsite diagnostic adequacy was established with fewer passes and definitive diagnosis was achieved in 85% of cases on first pass compared to 58% with the standard technique [61].

#### 3.1.3. Capillary/Slow Pull Technique

Another EUS-FNA technique is called the capillary/slow pull technique, in which the stylet is slowly retracted as FNA passes are being performed. This technique creates a slight negative pressure that increases aspiration of tissue. In one prospective study, the diagnostic ability of capillary vs. suction technique was compared in patients with solid pancreatic mass. High quality, adequate, and uncontaminated samples were obtained with the capillary technique. There was no significant difference in diagnostic accuracy of malignancy between capillary and suction techniques (90% vs. 90%, *p* = 1.00) [62].

#### 3.1.4. Wet Suction Technique

This technique is commonly used during EUS-FNA tissue acquisition. In this technique, the column of air is replaced with saline, and suction is applied to procure more tissue from the target lesion. In one RCT, standard FNA technique was compared with the wet suction technique using a 22-gauge FNA needle. Results showed that the wet suction technique yielded a significantly higher amount of cellularity in the cellblock compared to standard techniques [63].

Future research should focus on the benefit of using high negative pressure, wet suction, and capillary technique on specimen adequacy and yield of malignancy [64,65]. There are no well-defined guidelines on the optimal number of passes required to get a sufficient amount of tissue for cytologic and pathologic diagnosis. As procedure is completely operator dependent [66]. A recently published RCT compared patients undergoing EUS-FNA with or without ROSE for solid pancreatic masses [67]. All pancreatic lesions were included. Results showed that malignancy was detected with 92% sensitivity at four passes. Tumor size >2 cm was the only significant factor associated with positive cytology (odds ratio, 7.8; 95% CI, 1.9–31.6). In patients with tumor size >2 cm, four passes had a sensitivity of 93%, and six passes had a sensitivity of 82% for masses <2 cm without increase in sensitivity with further passes. This study suggested that in the presence of ROSE, at least four passes should be performed to achieve optimal sensitivity in pancreatic tumor size of >2 cm and six passes in masses with <2 cm. When on-site cytology results are inadequate or negative; EUS-FNB can be considered as an alternate method of sampling in patients with pancreas cancer. Although FNA needles are available in 25, 22, and 19-gauge, tissue acquisition with EUS-FNA of solid pancreas lesions are commonly performed using a 22 or 25-gauge needle. Common use of 25-gauge for EUS-FNA for pancreatic masses is supported from meta-analysis by Madhoun et al. that showed the 25-gauge needle has greater sensitivity compared to 22-gauge needles (pooled sensitivity 0.93 (95% CI 0.91–0.96) vs. 0.85 (95% CI 0.82–0.88)) [52].

### 3.2. EUS-FNB Needles and Designs

In recent years, EUS-FNB needles have generated a great deal of attention in the field of EUS. It is useful for obtaining core biopsy specimens, which is important for histologic or pathologic diagnosis. EUS-FNB has advantages over FNA in improving diagnostic yield in cases of prior negative diagnostic EUS-FNA (salvage approach), improving the assessment of tissue architecture and allowing for immunohistochemical stains (in cases of autoimmune pancreatitis, gastrointestinal stromal tumors, lymphoma, and metastases), and potentially, could avoid the use of ROSE and procedure time resulting in cost savings [64,66,68]. In addition, histologic tissue is important for evaluation of molecular markers and genomic profiling. Molecular profiling or mutational assays of tumor specimens could provide targeted therapies in patients with poor prognoses, like PDAC [69].

Recently published studies have shown success in tissue acquisition with FNB needles [70,71,72]. Three different needle sizes are available on the market, 19, 22, and 25-gauge, with designs such as reverse bevel needles (Pro-core, Cook Medical; Winston Salem, NC, USA), Franseen type needles (Acquire, Boston Scientific, Marlborough, MA, USA), and fork-tip needles (Shark Core, Medtronic, Minneapolis, MN, USA).

A multicenter study from China included 408 patients with solid pancreatic or abdominal masses. Diagnostic yield of EUS-FNB was significantly higher compared to EUS-FNA (91% vs. 80%, *p *= 0.01) [73]. However, recent meta-analysis has shown no significant difference between EUS-FNB and FNA with respect to diagnostic accuracy, sample adequacy, histology yield, and FNB was associated with fewer passes in obtaining diagnostic material [74]. In both of these studies, 22-gauge reverse bevel (Pro-core) and standard 22-gauge needles were compared.

In a retrospective case control study by Kandel et al., a total of 156 patients with solid tumors were included. All EUS-FNB cases were matched at 1:3 ratios by lesion site and needle gauge with FNA. Histology yield and number of passes to obtain the histology core was compared between two different needles. Results showed that EUS-FNB with a fork-tip needle (Shark Core) achieved higher histology yield compared to EUS-FNA (Boston scientific corp, Marlborough, MA, USA) (95% vs. 59%, *p *= 0.01), with fewer passes (2 vs. 4 passes, *p *= 0.01) [75]. This result was further confirmed by a multicenter study from Dimaio et al. as the fork-tip needle (Shark Core) had excellent pathologic diagnostic yield for pancreatic cancer with fewer passes [76].

Nayer et al. compared the diagnostic performance and yield for tissue acquisition from solid pancreas masses with two EUS-FNB needles: a reverse bevel needle (Pro-core) versus a novel fork-tip needle (Shark-Core). A total of 201 patients were included; opposing bevel (*n* = 101) and reverse bevel (*n* = 100). Results showed that the fork-tip needle had significantly higher sensitivity (90.1% vs. 71.1%, *p *= 0.006) and accuracy (92% vs. 74%, *p *= 0.006) for distinguishing benign solid pancreatic masses. Histology yield was significantly higher with the opposing bevel (Shark Core) needle compared to the reverse bevel (Pro-core) needle (99% vs. 87%, *p *= 0.002) [77]. A multivariate analysis, after controlling for needle gauge and site, did not show any significant difference in accuracy and sensitivity between the two groups.

Despite wide use of FNB needles in clinical practice, an ideal needle design is yet to be determined. As most PDAC are diagnosed by FNA with greater sensitivity, the perfect setting of FNB use is still an unanswered question.

### 3.3. Role of ROSE in EUS-Guided Tissue Acquisition

The main aim of ROSE is to give immediate response with regards to EUS-guided tissue acquisition at the time of procedure, such as specimen adequacy and content of the material present and reduction of passes necessary to achieve adequate diagnoses. This is a major limitation of EUS-FNA as diagnostic yield is totally dependent on ROSE results. One retrospective study of 182 patients with pancreatic mass who underwent EUS-FNA with and without ROSE showed that the ROSE arm was associated with fewer inadequate samples (1% vs. 12.6%), greater sensitivity (96% vs. 78%) and fewer passes [78]. However, a multicenter RCT showed no statistically significant difference with or without ROSE in the diagnostic yield of malignancy and specimen adequacy during EUS-FNA of pancreas mass. There were no significant differences in the number of repeat procedures, overall procedure time, accuracy, or adverse events, although fewer passes were required in the ROSE arm [79]. Also, no difference was observed between the two groups with respect to cytologic features, bloodiness, number of cells/side, and contamination.

Recently published studies have shown no meaningful effect of ROSE on the final diagnosis during EUS-FNB [64,72,80,81]. FNB sampling may eliminate the use of ROSE without loss of diagnostic accuracy when obtaining dedicated core tissue. However, it may still be helpful to those centers with low adequacy rates (<90%) and fewer experienced endoscopists [82]. New generation FNB needles yield more adequate tissue for pathologic and cytologic diagnosis. In addition, it provides an opportunity to make cellblocks and facilitate auxiliary studies, like molecular profiling, which is important in the era of precision medicine.

## 4. Role of EUS-Guided Tissue Acquisition in Precision Medicine

Individualized medicine refers to the delivery of custom-based treatment to patients. Precision therapy can be delivered with the help of disease specific biomarkers, clinical bioinformatics, precision methods, and regulations [83]. Optimal therapy can be provided in a similar fashion, microbial cultures may be used to target antibiotics [55]. This could potentially maximize the therapeutic goal of chemotherapy regimens (theranositic) and minimize drug-related adverse events. The goal should be to cure cancer, if not, at least to increase overall disease-free and progression-free survival.

EUS has the benefit of acquiring tissue directly from the target lesion without invasive surgery. For decades formalin-fixed, paraffin-embedded (FFPE) tissue blocks have been used for the genomic analysis of tissue obtained from surgical specimens. It is not known whether tissue obtained from EUS-FNA will be sufficient for theranostic studies. In one study, only 12.4% of positive cases had a cell block that was adequate for theranostic studies [84]. Adenocarcinoma was the common diagnosis (88%), but yielded fewer cell blocks. Some of the published literature has shown that liquid cytologic samples of residual FNA are excellent sources for future theranostic studies [85,86,87,88,89,90,91]. It is still unclear whether FNB offers better advantages over FNA for NGS. In one study, Choudhuri et al. assessed FNA and first generation core needle biopsies concurrently on liquid cytology. Results showed that FNA needles provided improved cellularity, higher tumor fractions, and better NGS metrics compared to core needles [87]. Future research should focus on whether liquid cytology or cell blocks of PDAC are better for performing theranostic studies. EUS-FNB may be very useful if FFPE specimens are being utilized to run future ancillary genomic analysis as dedicated core tissue can be obtained.

## 5. Organoid and Xenograft Development and Feasibility of Whole Exome Sequencing of Tumor DNA in Pancreas Cancer: Use of New Generation FNB Needles in the Era of Individualized Medicine

### 5.1. Organoid Development of Pancreas Tissue with EUS-FNB

PDACs like most cancers, changes and adapt in response to chemoradiotherapy. While genomics may offer a method to predict response to therapy (theranostic), it is imprecise. Direct assessment of response, such as how microbial colonies are tested against many antibiotics, would allow empiric determination of optimal therapy. Organoids are a promising new method to achieve this goal [92].

Organoids are three dimensional tumor models of individualized human PDAC that are typically developed from surgical specimens of resected tumors. Organoids retain full tumor characteristics of pancreas cancer, including stromal matrix, which is believed to be responsible for resistance to chemotherapy, and therefore, can be used for experimenting with personalized medicine compared to two-dimensional culture models. Since the majority of PDACs are diagnosed at an advanced stage, creation of human PDAC organoids from FNA/B needles would be an important advancement. A pilot study by Buscaglia et al. included 26 patients with solid pancreatic mass who came for EUS examination. EUS-FNA was performed with ROSE. After diagnosis was confirmed suggestive of PDAC with EUS-FNA, two additional passes with FNB needles (22G) were performed to create organoids. The main outcome of the study was successful creation of PDAC organoids by EUS-FNB [93]. PDAC was the final diagnosis in 23 patients, lymphoma in two, and recurrent PDAC in one. Organoid creation was successful in 85% patients. Of newly diagnosed PDAC, five of 23 (22%) patients underwent surgical resection. Creation of matched organoids was successful in two resected tumors, and in vitro drug sensitivity testing was successful in three organoids. No adverse events like pancreatitis, bleeding, or infection were noted. These data suggest that EUS-FNB tissue acquisition can be utilized for pancreatic cancer organoid development and in vitro drug testing which could be vital in PDAC patients.

### 5.2. Xenograft Development of Pancreas Tissue with EUS-FNB

Patient-derived xenografts (PDXs) have also gained attention in cancer research due to their potential use for personalized cancer therapy. PDXs are typically developed by transferring resected tumor tissues acquired through surgery into an immunocompromised mouse to stimulate human tumor biology in vivo. The need for bulk, surgically excised tissue is one of the major limitations of PDX models. PDX models allow for valuable information about tumor biology, identification of therapeutic agents, and preclinical screening and evaluation of drugs for various cancers [94]. The success rate of developing xenografts depends on the presence of sufficient cells to initiate new tumor formation [95,96]. We believe that new PDX models of pancreas cancer can be generated from the tissue acquired through new generation FNB needles at the time of routine diagnostic work up. There are only a few studies that have utilized minimally invasive EUS tissue acquisition technique for establishment of xenografts derived from pancreatic cancer patients. Hermans et al. developed a PDX model by transferring pancreas tumor tissue obtained by FNB [97]. All biopsies of untreated pancreatic carcinoma during EUS-FNB and surgery were collected and processed to generate PDXs. Tissue engraftment rate was 60%. Overall morphology, somatic mutation, and copy number of profiles of FNB-derived PDXs were conserved and very similar to the primary tumor. In the future, PDXs can be used for drug discovery, biomarker development, and tumor biology studies. It is feasible to develop PDXs using a EUS-FNB tissue acquisition technique; however, possible limitations include engraftment failure, high cost, and delay in engraftment of 6 months limiting its value in real-time disease management [98].

### 5.3. Feasibility of Whole Exome Sequencing of Pancreas Tissue with EUS-FNB

New generation FNB needles, fork-tipped designed (Shark Core), have been shown to provide better and adequate core tissue for histologic diagnosis with fewer passes compared to standard FNA needles [75,77]. With this advancement, it will be possible to obtain more DNA from the tumor tissue and carry out comprehensive whole exome sequencing of pancreas cancer. A large amount of DNA will facilitate preoperative genomic profiling and chemosensitivity testing. This will be a significant breakthrough in the field of precision medicine. Though several studies have been conducted to characterize the expression profiles of pancreas cancer using FNA specimens [99], the ability to perform mutational assays using FNB specimens has not been studied yet. Low quantity of specimens acquired from FNA specimens may preclude the precise determination of a tumor’s genetic status [84]. We hope that specimens obtained from new generation FNB needles will enable whole exome sequencing of pancreas tissue. In a prospective, ongoing, tandem RCT, Kandel et al. [100] included patients with solid pancreas mass who came as a part of routine clinical care. All patients underwent both conventional EUS-FNA and EUS-FNB (order randomized) with a minimum of one pass (with each needle) for cytology and histology, and second and third passes (one with each needle) for DNA and RNA extraction. All procedures were performed with ROSE. Sixty percent of lesions were PDAC. On first pass, histology yield (83% vs. 23%), tumor cellularity (45% vs. 10%), surface area of FFPE samples (57% vs. 7%), core length (10 cm vs. 2 cm), and adequacy for foundation medication assay (83% vs. 17%) was clearly greater with EUS-FNB compared to EUS-FNA. DNA sufficient for whole exome sequencing on the first pass for PDAC was obtained from 74% of EUS-FNB vs. 54% of EUS-FNA specimens. Thus, this ongoing trial showed greater DNA yield, which may guide precision therapy in the future for pancreas cancer.

### 5.4. Biomarkers Development

Until recently, no blood markers have been developed and validated for screening of pancreas cancer. Some efforts have been made to perform microRNA analysis from EUS-FNA specimens [101,102,103,104]. However, the process is technically difficult as dissection of these tissue specimens is not widely available, and the amount of DNA and RNA obtained from these specimens is relatively very low. 

In one study, EUS-FNB was used for tissue acquisition of PDAC for diagnosis (resectable and unresectable). There was about 86% resemblance between FNB and surgical specimens. S100A2/A4 on FNB biopsies was associated with poor prognosis, and loss of SMAD4 on preoperative cell blocks was strongly correlated with poorer outcome and increased risk of metastases [105,106]. Thus, EUS-FNB may guide evaluation for biomarkers without laser dissection of blocks, which may ultimately help clinicians identify which treatment, chemotherapy or surgery, will benefit which patient [106].

## 6. Conclusions

The advancement in EUS-guided tissue acquisition has increased hope in the field of precision therapy by combining organoid and biomarker development, drug testing and discovery, whole exome sequencing, and early detection methods of free circulating DNA. These developments have given us the opportunity to understand individual tumor biology and push the field one step closer to individualized medicine. 

## Abbreviations

ACG	American College of Gastroenterology
ASGE	American Society of Gastrointestinal Endoscopy
CEH-EUS	contrast-enhanced harmonic endoscopic ultrasound
CT	computed tomography
EUS	endoscopic ultrasound
EUS-E	endoscopic ultrasound elastography
pSR	parenchymal strain ratio
wSR	wall strain ratio
EUS-FNA	endoscopic ultrasound fine needle aspiration
EUS-FNB	endoscopic ultrasound fine needle biopsy
EUS-TTNFB	endoscopic ultrasound-guided through-the-needle forceps biopsy
FFPE	formalin-fixed paraffin embedded
MRI	magnetic resonance imaging.
NGS	next-generation sequencing
OCE	onsite cytological evaluation
PDAC	pancreatic ductal adenocarcinoma
PDX	patient-derived xenografts
RCT	randomized controlled trial
ROSE	rapid on-site evaluation
SEER	surveillance epidemiology and end results database
UEGW	United European Gastroenterology Week
nCLE	Needle-based confocal laser endomicroscopy
EUS-TTNFB	EUS-guided through-the-needle forceps biopsy
(ESGE)	European Society of Gastrointestinal Endoscopy
